# Long-term follow-up of stemless anatomic shoulder arthroplasty with a ceramic humeral head prosthesis: a multicenter study

**DOI:** 10.1016/j.jseint.2025.06.007

**Published:** 2025-07-03

**Authors:** Cormac Kelly, Géza Pap, Richard W. Nyffeler, Falk Reuther, Ulrich Irlenbusch

**Affiliations:** a The Hand and Upper Limb Unit, Robert Jones and Agnes Hunt Orthopaedic Hospital, Oswestry, Shropshire, UK; b Helios Park-Klinikum Leipzig, Leipzig, Germany; c Orthopädie Sonnenhof KLG, Bern, Switzerland; d DRK Kliniken Berlin Köpenick, Clinic for Trauma Surgery and Orthopaedics, Berlin, Germany; e Sports Clinic Erfurt, Erfurt, Germany

**Keywords:** Stemless shoulder prosthesis, Ceramic humeral head prosthesis, Total shoulder arthroplasty, Hemiarthroplasty, Radiolucent lines, Long-term prosthesis survival, Complications

## Abstract

**Background:**

Stemless anatomic total shoulder arthroplasty (aTSA) with ceramic implants have shown promising midterm clinical outcomes. However, long-term clinical data on ceramic humeral head prostheses are not available. We therefore evaluated the long-term clinical and radiographic outcomes, including implant survival and complication rates, of a stemless ceramic humeral head prosthesis in different shoulder pathologies.

**Methods:**

In this prospective, multicenter, observational study, patients underwent stemless aTSA using a ceramic humeral head prosthesis. We recorded Constant–Murley Scores (CSs), radiolucent lines (RLLs), complications, and long-term prosthesis survival.

**Results:**

We treated 238 patients (238 shoulders) with a stemless ceramic humeral head prosthesis. Clinical and radiographic outcomes were recorded from 120 shoulders at a median follow-up of 125.4 months, and complications from 229 shoulders. At final follow-up, CSs improved significantly from preoperative values (*P* < .0001). Although RLLs appeared both at the glenoid and humerus, osteolysis, wear, and aseptic loosening were rare (0.9%) and confined to the glenoid side; no aseptic loosening of the humeral component was noted. Prosthesis survival at 13 years reached 89.9% for all revisions and 90.8% for humeral component revision.

**Conclusion:**

Stemless aTSA with a ceramic humeral head prosthesis resulted in good CSs, a low number of clinically relevant RLLs, low incidences of glenoid osteolysis and aseptic loosening, no aseptic loosening of the humeral component, and high prosthesis survival rates in the long term. Moreover, nine out of ten prostheses remained in situ and were functional after 10 years, confirming the long-term success of this ceramic prosthesis.

Stemless shoulder arthroplasty relies on metaphyseal fixation at the humerus.^6^^,^^30^ Consequently, the humeral head position of a stemless shoulder prosthesis remains independent of the humeral shaft anatomy,^9^^,^^30^ and thus, theoretically, the head can always be placed in the correct anatomical position.^6^^,^^9^ Therefore, stemless shoulder arthroplasty is especially useful in patients with post-traumatic shoulder osteoarthritis (OA), fracture sequelae, or deformities of the metaphyseal region.^8^^,^^9^ In addition, using stemless shoulder prostheses in younger patients with high functional demands reduces the risk of periprosthetic fractures at the humeral shaft and enables a rather easy conversion to stemmed prostheses when needed.^9^

Several stemless shoulder implants are available today, which differ in design, articulating surface, surface coating, insertion technique, and bone contact area.^12^^,^^41^ One of the design innovations in shoulder arthroplasty is the use of ceramic humeral heads, with their potential of being used in stemless shoulder arthroplasty and hemiarthroplasty (HA).^23^ Ceramic heads have been successful in partial and total hip arthroplasty for a long time, where ceramic-on-polyethylene (PE) bearings have shown higher survival, lower revision, and lower PE wear rates than metal-on-PE bearings.^19^^,^^22^^,^^42^ However, the role of ceramic heads in shoulder arthroplasty is less studied due to the limited availability of ceramic implants.^23^ In fact, the prosthesis used in our study is the only ceramic stemless shoulder implant available on the market today featuring a ceramic humeral head and a two-peg all-PE glenoid component, and is compatible with both HA and anatomic total shoulder arthroplasty (aTSA) procedures. The ceramic-on-PE implant used in our study has shown less PE wear with it than its metal-on-PE counterparts, both in vitro and in patients, when combined with a PE glenoid component.^5^^,^^34^ Reduced PE wear after ceramic-on-PE stemless shoulder arthroplasty than metal-on-PE stemmed shoulder arthroplasty could potentially result in fewer glenoid radiolucent lines (RLLs) and less osteolysis on the humeral side.^5^

To date, only short- to midterm clinical and radiographic results are available for the stemless ceramic humeral head prosthesis used in this study, and these involve a limited number of patients and shoulder pathologies.^5^, ^6^, ^7^^,^^10^^,^^11^^,^^15^^,^^16^^,^^20^^,^^21^^,^^25^, ^26^, ^27^, ^28^, ^29^^,^^32^^,^^36^, ^37^, ^38^, ^39^ Thus, long-term clinical and radiographic data are needed to confirm the clinical profile this prosthesis and explore its potential of reducing PE wear-related complications, which will be especially valuable for younger patients who are likely to undergo revision surgery in their lifetime. We therefore evaluated the long-term clinical and radiographic outcomes of stemless aTSA with a ceramic humeral head prosthesis in different shoulder pathologies and recorded implant survival and complication rates.

## Materials and methods

This prospective, multicenter, observational study enrolled patients from five specialized shoulder centers (three in Germany, 1 in the United Kingdom, and 1 in Switzerland) between August 2009 and May 2012 who underwent HA and aTSA. The study involved patients from a previously published study.^25^

Clinical and radiographic examinations were carried out preoperatively and at 3 (radiographic examination only), 6, 12, 24, 48, 84, and 120 months; however, we only included patients with a minimum follow-up of 120 months in this study.

All patients gave written informed consent to participate in the study and agreed to the follow-up examination. The study received ethics committee approval from the Freiburger Ethik-Kommission International (number 012/1077), and all procedures were conducted in accordance with the Declaration of Helsinki.

**Figure 1:**
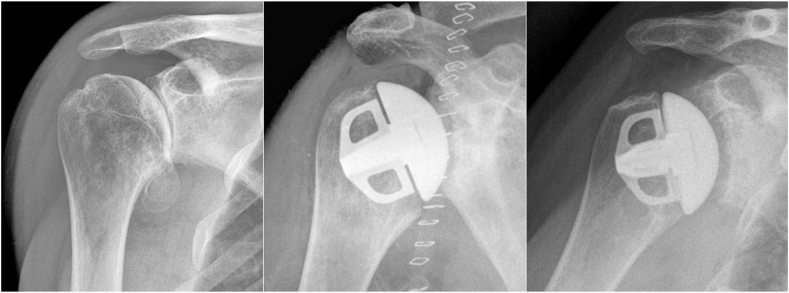
A typical aTSA case with the Affinis Short prosthesis. Radiographs taken preoperatively, immediately postoperatively, and at 10 years postoperatively. *aTSA*, anatomic total shoulder arthroplasty.

We operated on the patients in a beach chair position under general anesthesia using the deltopectoral approach in all shoulders. The subscapularis tendon was either transected, peeled off the lesser tuberosity, or detached from the humerus with a lesser tuberosity osteotomy. The technique was left to the surgeon's discretion. All patients received the Affinis Short stemless shoulder prosthesis (Mathys Ltd. Bettlach/Enovis, Switzerland) (Fig. 1), which features a four-wing humeral component design and a large-pored titanium structure with a calcium phosphate coating for uncemented fixation. We used it in combination with the Affinis Short Bionit ceramic head (ultrapure aluminum oxide with weight percentage of least 99.98%; Mathys Ltd. Bettlach/Enovis, Bettlach, Switzerland) and a cemented ultra–high–molecular–weight PE glenoid component (Mathys Ltd. Bettlach/Enovis, Bettlach, Switzerland). The cementation technique was left to the individual surgeon. The decision in favor of HA or aTSA was also left to the surgeon and influenced by the patient's age, bone quality, and the retroversion of the glenoid, as well as the condition of the rotator cuff and musculature.

**Figure 2:**
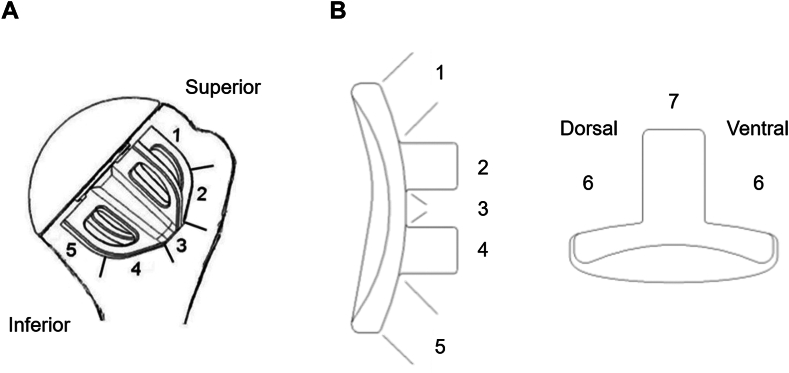
Schematics showing the radiographic zones at the proximal humerus (**A**) and glenoid (**B**).

Clinical assessment was conducted using the absolute and age- and gender-adjusted Constant–Murley Score (CS).^13^ Anteroposterior and lateral axillary radiographs were assessed by the local surgeon for the presence and width of RLLs around the humeral (five zones) and glenoid component (eight zones) in the anteroposterior and axillary view (Fig. 2). All complications and revisions were recorded.

### Statistical analysis

We reported descriptive statistical figures as means, medians, and ranges. Categorical data were presented as frequencies and percentages, and group comparisons for continuous variables were evaluated using the nonparametric Wilcoxon 2-sample test. We analyzed prosthesis survival using the Kaplan–Meier method, censoring patients at death or loss to follow-up. We defined final follow-up as the last date a clinical and/or radiographic evaluation was made. Confidence intervals were calculated using the log-log transformation. The log-rank test was used to test equality over strata. The level of significance was set at a *P* value of .05 (2-sided).

We performed all statistical analyses with the Statistical Analysis System, version 9.4 (SAS Institute Inc., Cary, NC, USA).

## Results

We treated 238 patients (238 shoulders: 144 in females and 94 in males) with a stemless shoulder prosthesis: 71 underwent HA and 167 aTSA. The mean age of the patients was 65.2 years (range, 30.4-87.1 years) at the time of surgery, and females were significantly older than males (mean age of 67.3 years vs. 62.0 years, *P* = .0001).

Patients were examined at a median follow-up of 125.4 months (range, 117.0-160.4 months). No significant differences in follow-up periods existed between the stemless HA and total shoulder arthroplasty (TSA) groups (125.7 vs. 125.1 months, *P* = .679). However, significantly more females were treated with stemless TSA than HA (75.7% vs. 61.7%, *P* = .021).

The main indication for stemless aTSA was primary OA (53.3%), followed by post-traumatic OA (19.2%), rheumatoid arthritis (10.8%), humeral head necrosis (10.2%), other indications (3.6%), fracture sequelae (2.4%), and cuff tear arthropathy (0.6%). For stemless HA, primary OA (38.0%) was followed by humeral head necrosis (25.4%), post-traumatic OA (15.5%), rheumatoid arthritis (9.9%), fracture sequelae (4.2%), other indications (4.2%), and cuff tear arthropathy (2.8%). Other indications included synovial chondromatosis, tumor, and post–childhood infection.

**Figure 3:**
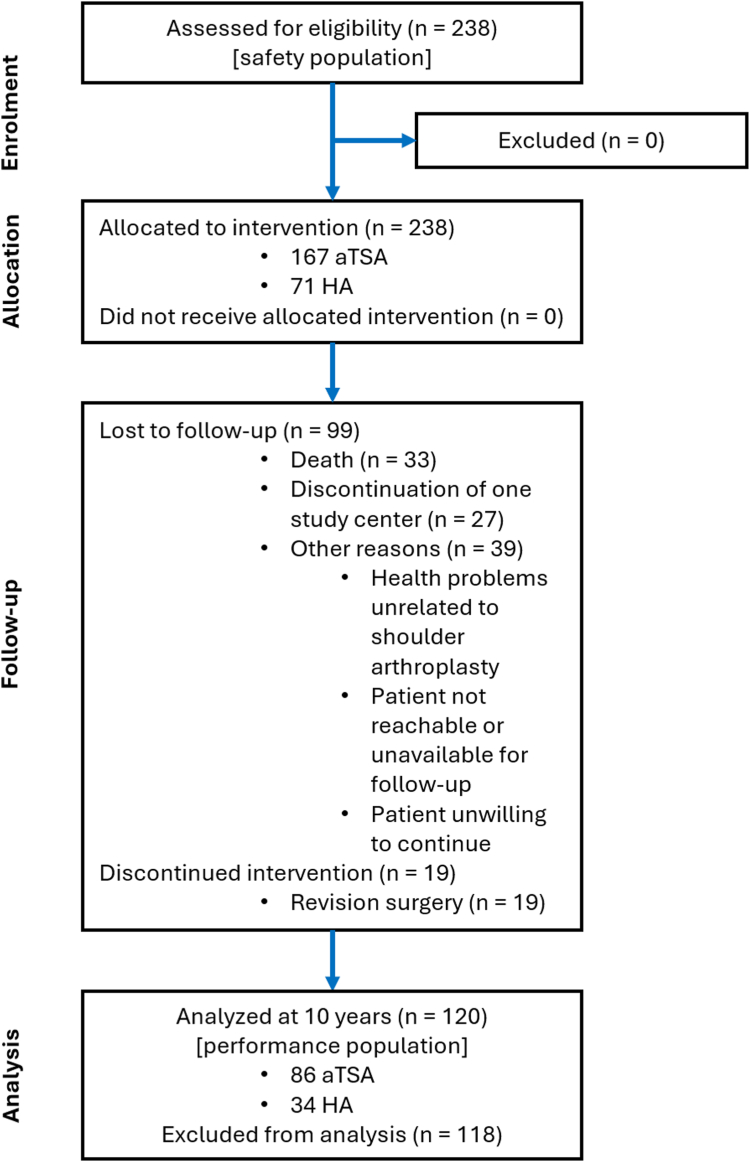
Flowchart showing the number of patients for enrollment, allocation, follow-up, and analysis. *HA*, hemiarthroplasty; *aTSA*, anatomic total shoulder arthroplasty.

The final clinical and radiographic analysis included 120 patients (34 HAs, 86 aTSAs); the remaining 118 patients were excluded because of an insufficiently long follow-up period owing to death, revision surgery, discontinuation of one study center, or other reasons (Fig. 3).

At the final follow-up examination, the absolute and age- and gender-adjusted CSs improved significantly from preoperative values (Table I) and were similar between patients who underwent stemless HA and TSA (absolute CS: *P* = .602; age- and gender-adjusted CS: *P* = .425). We noted that females had a lower mean postoperative absolute CS value than males: 65.0 vs. 78.2 (*P* < .0001). However, the difference in the improvements in absolute CSs from the preoperative to the final follow-up examination was comparable between females and males: 41.1 vs. 43.9 (*P* = .516). In addition, the gender-adjusted CS value was not significantly different between females and males at the final follow-up examination: 90.8 vs. 91.1 (*P* = .122).

**Table I:** Clinical scores of stemless shoulder arthroplasty.

Clinical outcome	HA	TSA
Absolute CS (points)		
Baseline	29.3 (5.0-52.0)	27.7 (8.0-78.0)
At final follow-up	72.8 (12.0-96.0)	69.5 (10.0-98.0)
*P* value∗	<.0001	<.0001
Age- and gender-adjusted CS (points)		
Baseline	35.9 (7.2-62.7)	35.9 (10.9-79.6)
At final follow-up	90.2 (14.4-122.9)	91.2 (12.5-133.3)
*P* value∗	<.0001	<.0001

*CS*, Constant–Murley Score; *HA*, hemiarthroplasty; *TSA*, total shoulder arthroplasty.

Reported as means with ranges shown in parentheses.

*P* values from paired *t*-test.

∗ Difference between baseline and final follow-up examination.

**Figure 4:**
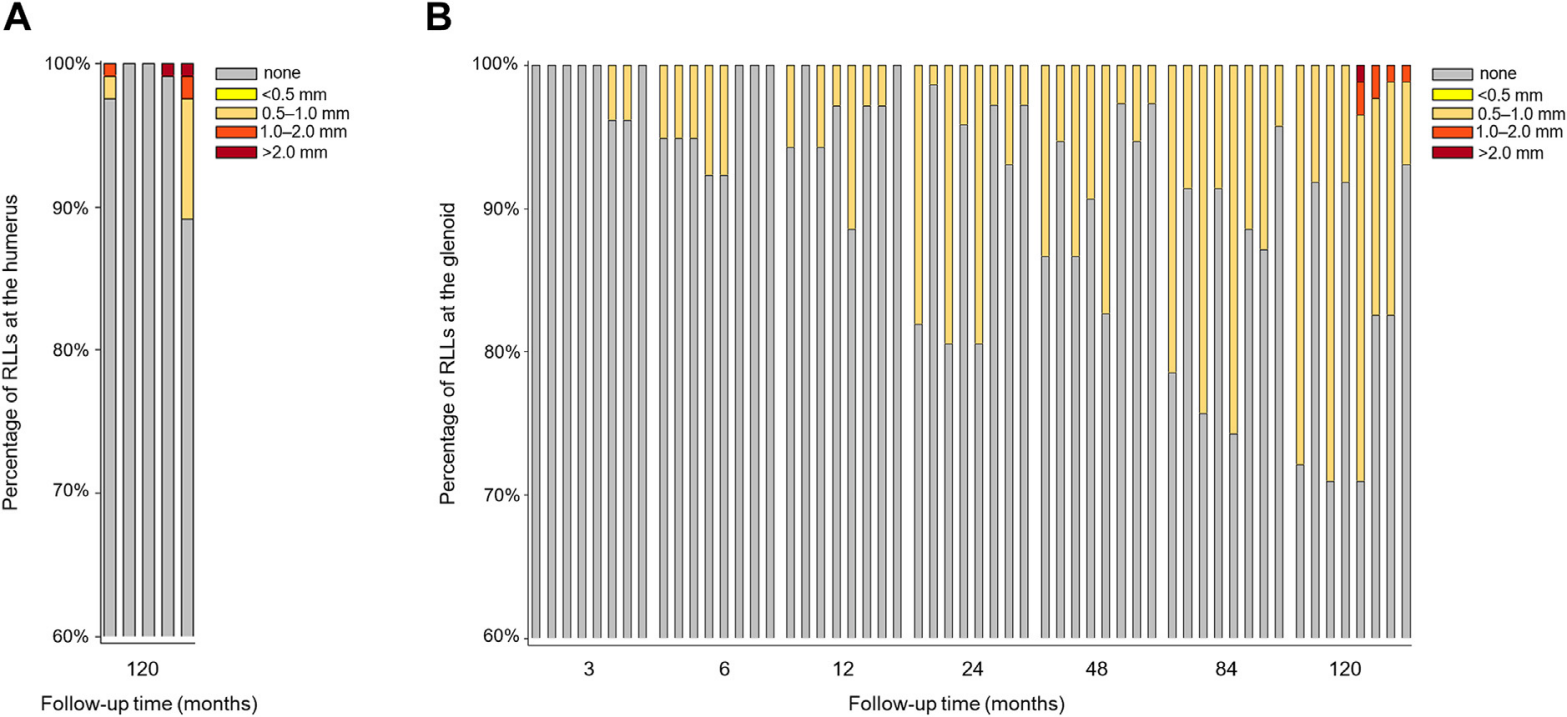
Bar graphs showing the percentage of RLLs in all zones in the humerus at 10 years (**A**) and in the glenoid over the follow-up period (**B**). Each bar represents 1 zone: zones 1-5 in the humerus and zones 1-7 in the glenoid (order of zones: 1-5, 6 dorsal, 6 ventral, 7). *RLLs*, radiolucent lines.

RLLs were observed both at the humerus and the glenoid. At the humerus, RLLs were seen in 28 (23.3%) humeri (zones 1-5) over the follow-up period, without significant differences between the stemless HA and TSA groups (*P* = .060). At the last follow-up examination, humeral RLLs of > 2 mm were recorded in two shoulders, once in zone 4 and once in zone 5 (Fig. 4, *A*).

At the glenoid, 53 of 86 (61.6%) shoulders treated with stemless TSA showed radiolucency (zones 1 to 7) over the follow-up period, and their presence gradually increased over time (Fig. 4, *B*). However, RLLs of >2 mm were absent until the last follow-up examination, at which time their presence was noted once in zone 5.

Complications were assessed from patients who had at least one clinical follow-up with nonmissing safety information, which amounted to 229 of 238 patients over the entire follow-up period; the remaining 9 patients had missing follow-up information and were therefore excluded. The overall median follow-up was 118.9 months (range, 0.1-160.4 months) for safety analysis and 142.2 months (range, 0.1-165.4 months) for prosthesis survival.

A total of 34 (14.8%) patients experienced one or more complications over the follow-up period, which included shoulder instability, rotator cuff avulsion, biceps tendon avulsion, and other complications such as arm or shoulder pain, subscapularis tendon rupture, and shoulder impingement, to name the most frequent ones. Complications specific to the ceramic head, such as ceramic fracture, were not observed. The overall complication rates were similar between patients undergoing stemless HA and TSA (14.9% vs. 14.8%, *P* = .983).

Revision surgery was carried out in 19 (7.9%) patients: 8 in the HA group and 11 in the aTSA group. Reverse total shoulder arthroplasty was used as a revision procedure in most cases. Prosthesis-related revisions were carried out in 11 patients (5 in the HA group and 6 in the aTSA group) and are listed in Table II. In the remaining 8 patients, revision surgery was performed for other reasons, such as secondary rotator cuff tear, proximal humeral fracture after a fall, or ongoing pain, among others. In addition, no revisions were recorded due to aseptic loosening of the humeral component.

**Table II:** Reasons for revision of stemless aTSA.

Patient age at index procedure (yr)/gender	Index procedure	Reason for revision	Time to revision (yr)	Revision procedure
53/M	HA	Glenoid erosion with pain	6.5	rTSA
80/F	HA	Glenoid erosion with pain	1.5	rTSA
47/F	HA	Persistent pain/suspected low–grade infection	0.2	Arthrodesis
64/M	HA	Rotator cuff insufficiency with glenoid erosion	6.4	rTSA
61/M	HA	Septic loosening of the humeral component	6.9	rTSA
51/M	TSA	Glenoid component wear	12.1	TSA
66/M	TSA	Infection	10.1	rTSA
70/F	TSA	Osteolysis and aseptic loosening of the glenoid component	8.7	HA with femoral head allograft
59/M	TSA	Posttraumatic dislocation of the glenoid with septic loosening of the humeral component	3.0	TSA
62/M	TSA	Septic loosening of the glenoid component	7.7	rTSA
72/F	TSA	Cuff failure instability and subluxation	7.3	rTSA

*aTSA*, anatomic total shoulder arthrplasty; *F*, female; *HA*, hemiarthroplasty; *M*, male; *rTSA*, reverse total shoulder arthroplasty; *TSA*, total shoulder arthroplasty.

**Figure 5:**
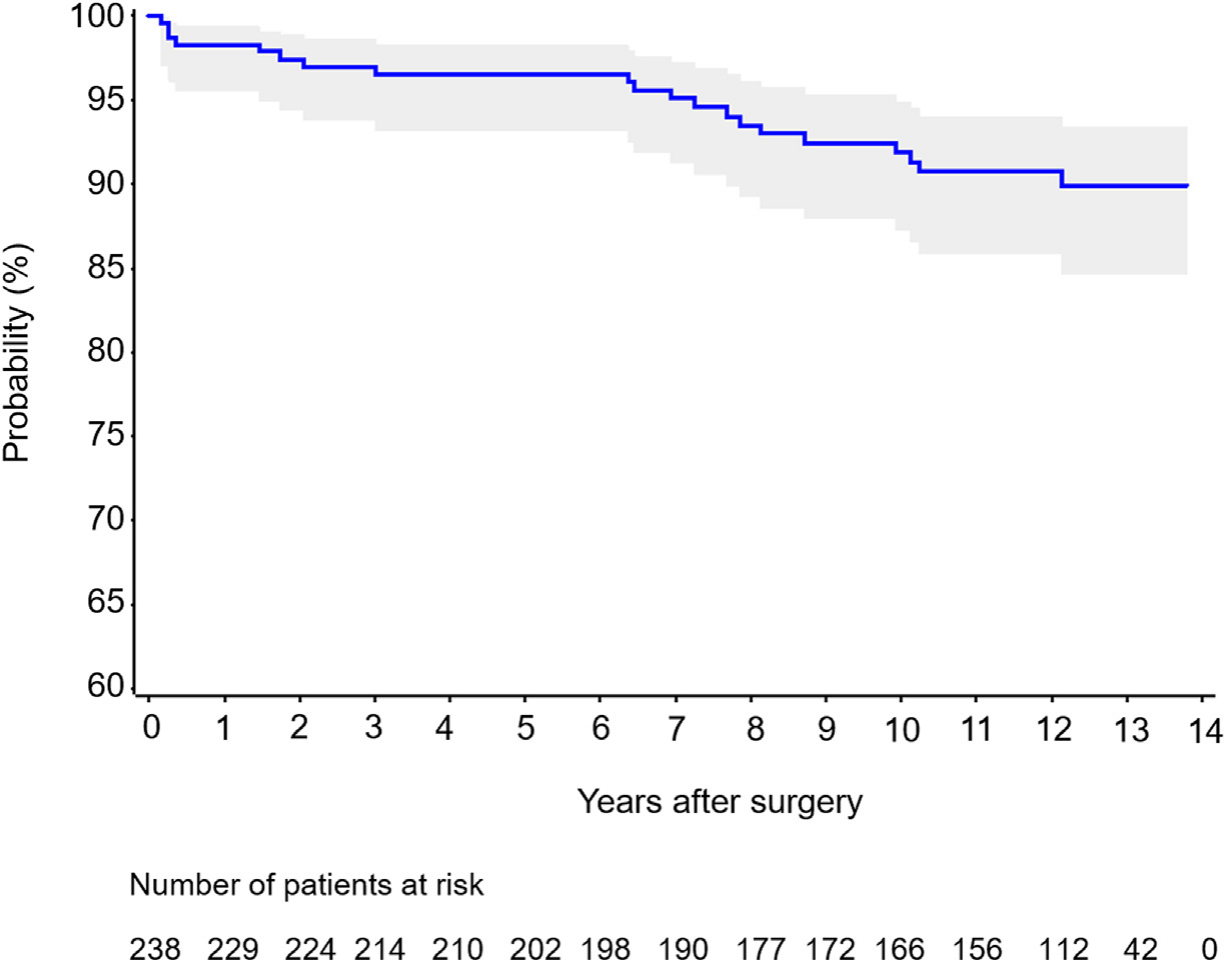
Kaplan–Meier prosthesis survival curve with revision for any reason. The shaded *gray* area represents upper and lower limits of the 95% confidence intervals.

Overall, prosthesis survival for all revisions, regardless of the reason, was 89.9% (95% confidence interval, 84.6% to 93.4%) at 13 years (Fig. 5) and similar in patients with stemless HA and TSA (86.7% vs. 91.2%, *P* = .234). The overall prosthesis survival for humeral component revision was 90.8% (95% confidence interval, 85.9%-94.0%).

## Discussion

Our study evaluated, for the first time, the long-term clinical and radiographic outcomes and prosthesis survival rates of the only available stemless ceramic shoulder prosthesis implanted in a large series of patients with different pathologies. Previously published studies on the same stemless shoulder prosthesis had only covered the short and midterm (<10 years), had a lower sample size than the present study, or included cases where glenohumeral OA was the main indication for shoulder arthroplasty (Table III).^5^, ^6^, ^7^^,^^10^^,^^11^^,^^15^^,^^16^^,^^20^^,^^21^^,^^25^, ^26^, ^27^, ^28^, ^29^^,^^32^^,^^36^, ^37^, ^38^, ^39^

**Table III:** Present and published clinical results with the Mathys Affinis Short stemless shoulder prosthesis.

Author, yr	Number of patients/procedures	Mean patient age	Indication	Follow-up	Type of anatomic shoulder arthroplasty	Clinical scores	Revision rate
Present study	238 patients/238 procedures 120 patients/120 procedures for the 10-yr clinical and radiographic follow-up	65.2 yr	HA: primary OA (38.0%), humeral head necrosis (25.4%), post-traumatic OA (15.5%), RA (9.9%), fracture sequelae (4.2%), other indications (4.2%), and cuff tear arthropathy (2.8%) TSA: primary OA (53.3%), post-traumatic OA (19.2%), RA (10.8%), humeral head necrosis (10.2%), other indications (3.6%), fracture sequelae (2.4%), and cuff tear arthropathy (0.6%)	Median: 125.4 mo	HA, TSA	Mean absolute CS: HA: 72.8 TSA: 69.5 Mean age- and gender-adjusted CS: HA: 90.2 TSA: 91.2	7.9%
Bell 2020^5^	23 patients/23 procedures	67.5 ± 7.8 yr	OA	Mean: 5.5 yr	TSA	Mean ASES score: 86.3 ± 10.8 Mean DASH score: 8.8 ± 10.4 Mean VAS for pain: 0.261 ± 0.541	Not reported
Bell 2014^6^	97 patients/97 procedures 12 patients/12 procedures at 2 yr	65.92 yr (for patients at 2 yr follow-up)	Primary OA	2 yr (mean not reported)	TSA	Mean CS: 85.75 Mean ASES score: 92.58 Mean DASH score: 5.94	1%
Berth 2016^7^	28 patients/28 procedures	68.57 ± 5.8 yr	Primary OA	180 d (mean not reported)	HA, TSA	Mean absolute CS: 54.8 ± 7.2 Mean age- and gender-adjusted CS: 73.3 ± 11.4	Not reported
Cardenas 2021^10^	1 patient/2 procedures	56 yr	Dysbaric osteonecrosis	2 yr	HA	CS right shoulder: 63 CS left shoulder: 75	Not reported
Chawla 2023^11^	70 patients/70 procedures	70 yr	Primary OA	Mean: 42.8 mo	TSA	Not reported	Not reported
Dixon 2025^15^	64 patients/75 procedures	Median age: 69 yr	Primary OA (89%) RA (7%) AVN (3%)	Median: 6.1 yr	TSA	Median OSS: 47	5.2%
Edwards 2023^16^	43 patients/43 procedures 30 patients/30 procedures at 5 yr	67 yr	Primary OA	5 yr (mean not reported)	TSA	Mean ASES score: 94.44 Mean VAS for pain: < 1	0%
Greis 2023^20^	73 patients/73 procedures	64.4 yr	Primary OA (78%) Posttraumatic OA (11%) Other indications (11%)	Median: 85.0 mo	HA, TSA	Mean CS: 78.8 ± 9.9	5%
Halm-Pozniak 2024^21^	80 patients/80 procedures	67.4 ± 11.2 yr	Primary OA	Mean: 92 ± 14 mo	TSA	Mean absolute CS: 71.6 ± 14.2 Mean age- and gender-adjusted CS: 98.56 ± 19.9 Mean DASH score: 14.9 ± 15.2	9%
Jordan 2019^25^	207 patients/207 procedures	64.8 yr	Primary OA (72.5%) Posttraumatic OA (9.7%) RA (7.2%) Fracture sequelae (6.3%) Humeral head necrosis (1.9%) Cuff tear arthropathy (1.4%) Other indications (1%)	Mean: 70.7 ± 17.2 mo	HA, TSA	Mean CS at 4 yr: 70.9 ± 18.5	6.3%
Jordan 2019^26^	35 patients/35 procedures	65.2 yr for patients with TSA 67.9 yr for patients with HA	RA	Mean: 22.7 mo for patients with TSA 34.1 mo for patients with HA	HA, TSA	Mean OSS: 32.4-36.4	6%
Karssiens 2021^27^	127 patients/141 procedures	68 yr	Primary OA (95%) Other indications (5%)	Mean: 3.7 yr	TSA	Mean OSS: 43.3	3.5%
Kasten 2021^28^	51 patients/51 procedures	66 ± 10 yr	Primary OA	Mean: 25.5 mo	TSA	Mean CS: 71-74	4%
Koch 2021^29^	29 patients/29 procedures	63.5 ± 11.7 yrr	Primary OA	1 yr (mean not reported)	TSA	Mean CS: 73.7 ± 13.6 Mean DASH score: 11.8 ± 12.1	Not reported
McMillan 2021^32^	62 patients/72 procedures	69 yr	Primary and secondary OA	Mean: 3.9 yr	TSA	Mean OSS: 45	6%
Rankin 2023^36^	96 patients/115 procedures	69 yr	OA (89.6%) RA (6.9%) Other indications (3.5%)	Median: 3.5 yr	TSA	Median OSS: 47	3%
Raval 2023^37^	51 patients/51 procedures	70.2 yr	OA	Mean: 5.5 yr	TSA	Mean OSS: 44	2%
Simon 2022^38^	44 patients/44 procedures	77.33 ± 1.97 yr	OA	2 yr (mean not reported)	TSA	Mean CS: 75.20 ± 11.41 Mean ASES score: 90.58 ± 9.88 Mean DASH score: 10.42 ± 10.65 Mean VAS for pain score: 0.29 ± 0.85	0%
Simon 2022^39^	74 patients/74 procedures	68.1 ± 7.1 yr	OA	5 yr (mean not reported)	TSA	Mean ASES score: 94.28 ± 7.44 Mean VAS for pain score: 0.41 ± 1.15	1%

*ASES*, American Shoulder and Elbow Surgeons; *CS*, Constant–Murley Score; *DASH*, Disabilities of Arm, Shoulder and Hand; *HA*, hemiarthroplasty; *OA*, osteoarthritis; *OSS*, Oxford Shoulder Score; *RA*, rheumatoid arthritis; *TSA*, total shoulder arthroplasty; *VAS*, visual analogue scale.

Our long-term results with this prosthesis revealed good clinical outcomes and minimal PE wear-related radiographic findings and complications, and were comparable to those documented for other stemless shoulder prostheses used for similar indications.^1^^,^^2^^,^^4^^,^^14^^,^^17^^,^^24^^,^^30^^,^^31^ First, the mean absolute CSs in our study were in line with or higher than those found in the literature on stemless shoulder arthroplasty for both HA (ranging from 62 to 67) and TSA (ranging from 63 to 69) after at least 9 years of follow-up.^24^^,^^30^ In addition, the mean absolute CSs were not significantly different between the stemless HA and TSA groups in these studies,^24^^,^^30^ which was also reflected in our results. However, a direct comparison between the effectiveness of HA vs. TSA could not be made from the present study due to its nonrandomized setup. Interestingly, we observed a lower mean absolute CS in females than males at the final follow-up examination; however, the improvements in CSs were comparable. Moreover, the difference in CS values disappeared when adjusted for gender. Since significantly more females were treated with stemless aTSA than HA, we also compared the age- and gender-adjusted CSs in the two groups to rule out these two potential confounding factors and found no significant differences at the final follow-up examination. Second, our prosthesis survival rates were consistent with long-term values documented in the literature.^30^^,^^31^ Last, the overall revision rate of 7.9% in our study was substantially lower than the rates observed with other stemless shoulder prostheses over a similar follow-up period (ranging from 9.7% to 15.7%).^1^^,^^3^^,^^30^

One study showed that the stemless ceramic-on-PE prosthesis used in our study led to fewer glenoid RLLs and less humeral osteolysis in the midterm than a stemmed metal-on-PE prosthesis.^5^ Although we noted that the percentage of RLLs gradually increased on the glenoid side over time, the clinically relevant RLLs, ie, those of >2 mm width,^18^^,^^33^^,^^40^ occurred rarely (2.5%) in the long term. Moreover, complications potentially related to increased radiolucency, such as osteolysis, wear, and aseptic loosening, were rare (0.9%) and confined to the glenoid component. These radiographic results were superior to those reported for other stemless prostheses, where PE wear of the glenoid component was seen in up to 45.5% of stemless TSAs with a metal-on-PE interface and glenoid component loosening noted in 11.4% of cases after more than 10 years.^30^ The ceramic-on-PE articulation, which is known to be associated with less PE wear than metal-on-PE combinations,^5^ could be one of the several reasons contributing to this outcome. On the humeral side, no aseptic loosening of the humeral component was seen in our present long-term study, despite the bone changes on the medial calcar attributing to stress shielding in the midterm.^25^ Similarly, authors of another study with the same stemless ceramic-on-PE prosthesis reported most stress shielding in the medial calcar region in the midterm; however, stress shielding neither resulted in radiolucency nor negatively affected clinical outcomes.^36^ Another recently published study found no humeral radiolucency in the midterm with the same stemless prosthesis used in our study.^15^

Hypersensitivity is known to occur with the use of metal implants and can cause implant failure.^35^ In particular, incidence of nickel sensitivity has been reported in 11.4% of cases, followed by cobalt in 2.7% and chromium in 1.8% of cases.^35^ None of the patients in our study reported hypersensitivity or allergic reaction to the ceramic-on-PE implant, unlike with metal shoulder implants, where hypersensitivity has been reported in nearly 10% of patients at a mean follow-up of 45 months.^35^ In addition, we observed no complications specific to the ceramic humeral head over the entire follow-up period.

Notable strengths of our study are its large sample size, multicenter setup, inclusion of different shoulder indications, and long-term follow-up. In addition, this is the first study reporting long-term results of stemless aTSA with a ceramic humeral head prosthesis. Nevertheless, typical limitations associated with a multicenter study, such as variable surgical techniques and surgeon experiences, remain, even if the surgeons involved in this study are experienced and regularly perform shoulder arthroplasties. However, we lack a control group treated with a different stemless prosthesis for direct comparison. Future studies with appropriate controls will be needed to compare the results of this study with other contemporary stemless shoulder prosthesis. Another limitation is the high loss of patients from our midterm study^25^ to the present study, mainly due to difficulty in following up the patients at one center and the impact of the COVID-19 pandemic, which could also have led to loss of reported revision cases.

## Conclusion

Stemless aTSA with a ceramic humeral head prosthesis resulted in good CSs, a low number of clinically relevant RLLs, low incidences of glenoid osteolysis and aseptic loosening, no aseptic loosening of the humeral component, and high prosthesis survival rates in the long term. Moreover, nine out of ten prostheses remained in situ and were functional after 10 years, confirming the long-term success of this ceramic prosthesis. These results will be valuable for patients deemed suitable for aTSA, especially younger patients where revision surgery will likely be needed during their lifetime.

## Acknowledgment

The authors thank Dr. Dominik Pfluger at numerics data GmbH for statistical analysis, Mathys Ltd./10.13039/100022327Enovis for partially funding this study, and Medical Minds GmbH for providing medical writing and editorial support.

## Disclaimers:

Funding: The study was partially funded by Mathys Ltd. Bettlach/Enovis to support statistical analysis through an independent consultant, medical advisor contracts and travel expenses for C. Kelly, G. Pap, R.W. Nyffeler, F. Reuther, and U. Irlenbusch, as well as medical writing and editorial support from a medical writing agency.

Conflicts of interest: The authors, their immediate families, and any research foundation with which they are affiliated have not received any financial payments or other benefits from any commercial entity related to the subject of this article.

## Supplementary data

STROBE Checklist
