# Supplementary material for: Long-term follow-up of stemless anatomic shoulder arthroplasty with a ceramic humeral head prosthesis: a multicenter study
Source: JSES Int. 2025 Jul 3;9(6):2044–52. doi: 10.1016/j.jseint.2025.06.007 (PMC12828186; doi:10.1016/j.jseint.2025.06.007)
Supplement: STROBE Checklist [file mmc1.pdf]

STROBE Statement—checklist of items that should be included in reports of observational studies

	Item No.	Recommendation	Page No.	Relevant text from manuscript
**Title and abstract**	1	(*a*) Indicate the study's design with a commonly used term in the title or the abstract	1	Line numbers 2, 10
		(*b*) Provide in the abstract an informative and balanced summary of what was done and what was found	2, 3	Line numbers 5–27
**Introduction**				
Background/rationale	2	Explain the scientific background and rationale for the investigation being reported	3, 4	Line numbers 33–62
Objectives	3	State specific objectives, including any prespecified hypotheses	4	Line numbers 62–65
**Methods**				
Study design	4	Present key elements of study design early in the paper	5	Line number 67
Setting	5	Describe the setting, locations, and relevant dates, including periods of recruitment, exposure, follow-up, and data collection	5	Line numbers 67–70
Participants	6	(*a*) *Cohort study*—Give the eligibility criteria, and the sources and methods of selection of participants. Describe methods of follow-up<br>*Case-control study*—Give the eligibility criteria, and the sources and methods of case ascertainment and control selection. Give the rationale for the choice of cases and controls<br>*Cross-sectional study*—Give the eligibility criteria, and the sources and methods of selection of participants	5	Line numbers 71–73
		(*b*) *Cohort study*—For matched studies, give matching criteria and number of exposed and unexposed<br>*Case-control study*—For matched studies, give matching criteria and the number of controls per case	n/a	n/a
Variables	7	Clearly define all outcomes, exposures, predictors, potential confounders, and effect modifiers. Give diagnostic criteria, if applicable	6	Line numbers 91–95
Data sources/ measurement	8*	For each variable of interest, give sources of data and details of methods of assessment (measurement). Describe comparability of assessment methods if there is more than one group	6	Line numbers 91–95
Bias	9	Describe any efforts to address potential sources of bias	5, 6	Line numbers 78–95
Study size	10	Explain how the study size was arrived at	5	Line numbers 69–70

Continued on next page

Quantitative variables	11	Explain how quantitative variables were handled in the analyses. If applicable, describe which groupings were chosen and why	5	Line numbers 91–95
Statistical methods	12	(*a*) Describe all statistical methods, including those used to control for confounding	6	Line numbers 97–106
		(*b*) Describe any methods used to examine subgroups and interactions	6	Line numbers 97–99
		(*c*) Explain how missing data were addressed	6	Line numbers 99–102
		(*d*) *Cohort study*—If applicable, explain how loss to follow-up was addressed<br>*Case-control study*—If applicable, explain how matching of cases and controls was addressed<br>*Cross-sectional study*—If applicable, describe analytical methods taking account of sampling strategy	6	Line numbers 99–101
		(*e*) Describe any sensitivity analyses	n/a	n/a
**Results**				
Participants	13*	(a) Report numbers of individuals at each stage of study—eg numbers potentially eligible, examined for eligibility, confirmed eligible, included in the study, completing follow-up, and analysed	7, 8	Line numbers 108–110, 123–125, 144–148
		(b) Give reasons for non-participation at each stage	7, 8	Line numbers 123–125, 144–147
		(c) Consider use of a flow diagram	n/a	n/a
Descriptive data	14*	(a) Give characteristics of study participants (eg demographic, clinical, social) and information on exposures and potential confounders	7	Line numbers 109–111, 114–115
		(b) Indicate number of participants with missing data for each variable of interest	7, 8	Line numbers 123–125, 144–147
		(c) *Cohort study*—Summarise follow-up time (eg, average and total amount)	7, 8	Line numbers 112, 147–148
Outcome data	15*	*Cohort study*—Report numbers of outcome events or summary measures over time	7, 8, 9	Line numbers 126–143, 149–165
		Case-control study—Report numbers in each exposure category, or summary measures of exposure	n/a	n/a
		Cross-sectional study—Report numbers of outcome events or summary measures	n/a	n/a
Main results	16	(*a*) Give unadjusted estimates and, if applicable, confounder-adjusted estimates and their precision (eg, 95% confidence interval). Make clear which confounders were adjusted for and why they were included	n/a	n/a
		(*b*) Report category boundaries when continuous variables were categorized	7, 8, 9	Line numbers 116–122, 126–143, 149–165
		(*c*) If relevant, consider translating estimates of relative risk into absolute risk for a meaningful time period	n/a	n/a

Continued on next page

Other analyses	17	Report other analyses done—eg analyses of subgroups and interactions, and sensitivity analyses	n/a	n/a
**Discussion**				
Key results	18	Summarise key results with reference to study objectives	10	Line numbers 167–172
Limitations	19	Discuss limitations of the study, taking into account sources of potential bias or imprecision. Discuss both direction and magnitude of any potential bias	12	Line numbers 220–228
Interpretation	20	Give a cautious overall interpretation of results considering objectives, limitations, multiplicity of analyses, results from similar studies, and other relevant evidence	13	Line numbers 230–234
Generalisability	21	Discuss the generalisability (external validity) of the study results	13	Line numbers 234–236
**Other information**				
Funding	22	Give the source of funding and the role of the funders for the present study and, if applicable, for the original study on which the present article is based	Title page	Line numbers 20–26

*Give information separately for cases and controls in case-control studies and, if applicable, for exposed and unexposed groups in cohort and cross-sectional studies.

Note: An Explanation and Elaboration article discusses each checklist item and gives methodological background and published examples of transparent reporting. The STROBE checklist is best used in conjunction with this article (freely available on the Web sites of PLoS Medicine at http://www.plosmedicine.org/, Annals of Internal Medicine at http://www.annals.org/, and Epidemiology at http://www.epidem.com/). Information on the STROBE Initiative is available at www.strobe-statement.org.
